# Coronary artery calcium scoring using virtual vs. true non-contrast images from ultra-high-resolution and normal-resolution photon-counting coronary CT angiography

**DOI:** 10.1016/j.ejro.2026.100795

**Published:** 2026-07-04

**Authors:** Sophia Engel, Iram Shahzadi, Nina Pauline Haag, Roman Johannes Gertz, Julius Henning Niehoff, Alexey Surov, Jan Borggrefe, Jan Robert Kroeger

**Affiliations:** a Ruhr University Bochum, Johannes Wesling Hospital, Department of Radiology, Neuroradiology and Nuclear Medicine, Germany; b University of Cologne, Faculty of Medicine and University Hospital Cologne, Department of Radiology, Germany

**Keywords:** Arteries/ Aorta, Cardiac, CT-Angiography, Screening, Calcifications

## Abstract

**Objective:**

To validate the reliability of Coronary Artery Calcium Scoring (CACS) by using virtual non-contrast (VNC) images reconstructed from contrast-enhanced photon-counting coronary CT angiography (CCTA) versus true non-contrast (TNC) images, and to assess the impact on plaque burden classification.

**Materials and methods:**

239 patients who underwent contrast-enhanced photon-counting CCTA with TNC imaging were included. Agatston score was calculated on both (VNC and TNC) datasets. Scores ≤ 0.9 were rounded to 0. Patients were divided into plaque groups according to the CAD-RADS 2.0 classification system. Borderline cases (P0 vs. P1) were reassessed by two radiologists with blinded adjudication.

**Results:**

Median Agatston scores showed no significant difference (TNC: 14.2 [range: 0.0–2951.2] vs. VNC: 8.0 [range: 0.0–3069.3]; *p* = 0.54). Correlation was excellent (intraclass correlation coefficient, 0.97 [95% CI: 0.97, 0.98]. Robust linear regression revealed a significant relationship (intercept = 3.27 [95% CI: 1.28, 5.87]; slope = 0.97 [95% CI: 0.97, 0.98]; *p* < 0.001). Despite the excellent correlation, a significant difference in plaque classification was observed (*p* = 0.005). Classification was consistent in 160/239 (67%; *κ* = 0.88) patients. After a consensus reading, the agreement improved to 205/239 patients (86%; κ = 0.95).

**Conclusion:**

Agatston scores derived from TNC and VNC images show no significant differences and excellent consistency. However, the agreement for plaque classification is limited, preventing VNC images from directly substituting for TNC images for this purpose. While a subjective reading significantly improved agreement, further research is required before clinical implementation.

## Highlights

• PureCalcium shows excellent correlation with true non-contrast images regarding Agatston scores.
• Differences were revealed in plaque burden classification, especially for lower scores.
• Subjective reading improved plaque burden classification to almost perfect agreement.
• Radiation exposure could be significantly reduced by omitting a true non-contrast scan.
• Further research is needed before PureCalcium can be used in clinical practice.

## 1 Introduction

Invasive coronary angiography was long regarded as the standard method for diagnosing coronary heart disease (CHD). Recently, non-invasive diagnostics have gained prominence with Coronary CT Angiography (CCTA) now recommended as the primary diagnostic method in the current guidelines of the European and North American cardiology societies [1], [2]*.*

CCTA alone cannot provide accurate information about coronary artery calcium (CAC) scores, as contrast media and calcification are not always distinguishable on conventional CT [3]. Therefore, an additional true non-contrast (TNC) CT must be performed as part of a CCTA protocol, which results in additional radiation exposure for the patient [3]. With the advent of the new photon-counting CT (PCCT), it is now possible to generate spectral data of the coronary vessels [2]. This technology enables the generation of virtual non-contrast (VNC) CT images using a specialized algorithm (PureCalcium), thereby avoiding additional radiation exposure [3], [4].

The PureCalcium algorithm has only been used in four studies to date [3], [4], [5], [6]. A recent study by Haag et al. [5] has shown minimal differences between the conventional method and the PureCalcium algorithm, which consequently leads to a potential radiation dose reduction when using the PCCT. In the previous software version (CT CAS (CT Calcium Scoring), syngo.via VA50, Siemens Healthineers) applied in their study, however, technical limitations remained, as it was not possible to calculate PureCalcium-based virtual non-contrast (VNC_PureCalcium_) images from images with ultra-high image resolution. Notably, these ultra-high-resolution examinations were primarily performed in patients with a high calcium load on the coronary arteries, which resulted in a shift of Agatston scores towards lower values compared to the overall collective. The subsequent software version (CT CAS, syngo.via VB60, Siemens Healthineers) allows for CCTA examinations with simultaneous acquisition of ultra high-resolution (UHR) image data and spectral image data, thereby allowing iodine suppression and the reconstruction of VNC_PureCalcium_ images.

The purpose of the current study is to verify the results and the reliability of the VNC_PureCalcium_ images for a new collective with higher Agatston scores using the newer VB10 software generation. Specifically, we investigated whether using VNC_PureCalcium_ images would lead to clinically relevant patient misclassification. In the context of this collective, it was also evaluated whether there are any differences in the performance of the PureCalcium algorithm approach depending on the scan protocol used, in particular, UHR vs. normal resolution scans. This would be a further decisive step towards eliminating the need for dedicated non-contrast images in the future and thus ensuring lower radiation exposure for patients.

## 2 Materials and methods

### 2.1 Study design and patients

**Fig. 1:**
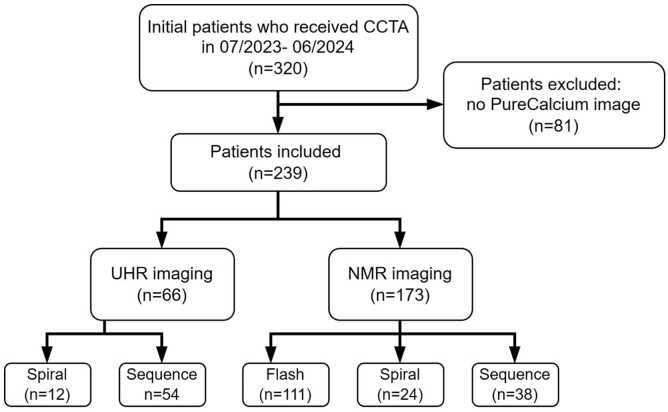
Flow Chart shows number of patients undergoing coronary CT angiography (CCTA) between July 2023 and June 2024 and their imaging.

This retrospective, single-center study was conducted in accordance with the guidelines of the Declaration of Helsinki and received approval from the institutional review board. The patient database was searched for adult patients who had undergone CCTA on the PCCT with the latest software version VB10 during the period from July 2023 to June 2024. From this collective, the patients who received an examination with spectral image information were identified. Finally, 239 patients were included in the study. [See Fig. 1].

### 2.2 Scan protocol

All examinations were performed on a PCCT system (Naeotom Alpha, VB10; Siemens Healthineers). The TNC (true non-contrast) scans are acquired using a high-pitch-scan (dual source).

The choice of CCTA scan mode depends on several factors. First, the TNC scan is evaluated for relevant calcifications or the presence of a coronary stent. If neither is present, a scan in Normal Resolution Mode (NRM) is performed. Depending on the heart rate (HR) and rhythm, a flash (HR<65), sequence (HR 65–90), or spiral (HR >90 and/ or arrhythmia) scan is used. If there is a relevant calcification or a stent present, UHR with spectral image data or normal UHR mode is performed. UHR imaging with spectral image data is only performed if a breath-hold of 15 s is possible for the patient. Depending on the heart rate and rhythm, a Sequence UHR scan with spectral image data (HR< 80) or a Spiral UHR scan with spectral image data (HR> 80 and/ or arrhythmia) is used. If a breath-hold of 15 s is not possible, a normal UHR mode is performed. The sequence UHR (HR <90) or spiral UHR (HR >90 or arrhythmia) scan is used.

Tube voltage was 120 or 140 kV, depending on the patient's constitution, selected automatically by the CT scanner. Automatic tube current modulation was used with an image quality level of 19 for TNC scans and 64 for the CCTA.

Up to 15 mg of metoprolol (1 mg metoprololtartrate/mL, Beloc; Recordati Pharma) was injected intravenously for heart rate reduction if not contraindicated. Additionally, if.

not contraindicated, 0.4 mg of nitroglycerin (Glyceroltrinitrat, G. Pohl-Boskamp) was given sublingually [5].

### 2.3 Image reconstruction

Image Reconstruction in axial orientation was performed using a 512 × 512 image matrix and soft body kernel. According to the predefined manufacturer-specific reconstruction settings of true non-contrast Images were reconstructed with 3 mm slice thickness using a Qr36 kernel, while PureCalcium-based virtual non-contrast images were derived from contrast-enhanced CCTA datasets and reconstructed using Qr40 kernel with quantum iterative reconstruction (QIR) level 3 at 2 mm slice thickness [5]. A more detailed description of the PureCalcium reconstruction algorithm was published by Emrich et al. [4].

### 2.4 Calcium scoring

Calcium scoring was carried out retrospectively using post-processing software (CT CAS, syngo.via VB60, Siemens Healthineers) with which semi-automated calcium scoring was performed.

A structure was automatically identified as calcium if it had a minimum size of 0.5 mm² and a density of at least 130 HU. These calcifications were then automatically assigned to the respective coronary arteries. This assignment was adjusted manually if necessary.

Agatston scores were calculated automatically on both TNC and corresponding VNC_PureCalcium_ datasets. According to the predefined software workflow. The determination was carried out on the standard images of the non-contrast scan in 3 mm slice thickness and on the corresponding PureCalcium images of 2 mm slice thickness. Agatston scores ≤ 0.9 were rounded to 0. In addition, the patients were divided into clinically relevant plaque groups, which were sorted according to the CAD RADS 2.0 classification system (P0 = 0 [none]; P1 = 1–100 [mild]; P2 = 101–300 [moderate]; P3 = 301–999 [severe]; P4 > 1000 [extensive]) [7].

For patients with a classification discrepancy between P0 and P1, two radiologists, with 4 years and 10 years of experience in cardiac radiology, independently analyzed the CCTA datasets in a blinded reading to determine whether no or minimal calcium was present. Only in cases of persistent discrepancy after blinded reassessment, a second consensus reading was performed for final adjudication. [see Fig. 1].

Finally, the radiation dose of the scans was recorded, and the potential radiation dose reduction if a non-contrast scan is not used was calculated.

### 2.5 Statistical analysis

Continuous variables were expressed as means ± standard deviation or median with Interquartile range. The normality of data was analyzed using a histogram and a Q-Q plot. The Wilcoxon signed-rank test (with the Pratt method to account for pairwise differences of 0) was applied to determine the statistical significance of the differences in the Agatston scores between TNC and VNC_PureCalcium_ scans and their corresponding plaque classification [8].

Additionally, to evaluate the equivalence of paired measurements, repeatability limits were calculated using an established model by Chung et al. [9]. The repeatability limit (RL) was defined by the following equation:

$$\mathrm{RL}=\;\left(0.17\times M\right)+\;\left(5.00\times \sqrt{M}\right)+1.08$$

where *M* represents the mean Agatston score, calculated as:

$$M=\;\frac{{\mathrm{Agatston}\;\mathrm{score}}_{\mathrm{TNC}}+{\mathrm{Agatston}\;\mathrm{score}}_{\mathrm{PureCalcium}}}{2}$$

The proportion of measurements falling within the repeatability limit was subsequently determined to assess equivalence.

The agreement between TNC and VNC_PureCalcium_ Agatston scores was assessed using the Bland-Altman analysis with 95% limits of agreement and intraclass correlation coefficient (ICC). Due to the heteroscedastic distribution of Agatston scores, the Bland-Altman plot and limits of agreement were calculated using the quantile regression method [9]. The limits of agreement from Equation 1 were also plotted for comparison [9]. ICC was interpreted as follows: less than 0.50 (poor), 0.50 to less than 0.75 (moderate), 0.75 to less than 0.9 (good), and 0.9–1.0 (excellent). Given the non-normal distribution and the presence of extreme values in the Agatston score, the relationship between TNC and VNC_PureCalcium_ was explored by fitting a robust linear regression model to predict TNC from VNC_PureCalcium_ measurements.

The agreement of plaque classification categories between TNC and VNC_PureCalcium_ was analyzed using weighted Cohen's kappa (*κ*) statistics. Kappa values were interpreted as follows: 0.4 or less (poor), 0.41–0.6 (moderate), 0.61–0.8 (good), and 0.81–1.0 (excellent agreement). The percentages of the dose-length product from TNC and the dose-length product of the entire examination, TNC and CCTA scan, were calculated on a per-patient basis to assess radiation doses. Subsequently, the mean and SD of these percentages were calculated.

All statistical tests were two-sided. P-values are reported as exact values to two significant figures, with values below 0.001 reported as *p* < 0.001. Confidence intervals are reported at the 95% level throughout. Statistical significance was defined as *p* < 0.05. All statistical analyses were performed using Python (version 3.12.4) using pandas, SciPy (v1.11.3), and statsmodels (v0.14.6) for statistical tests.

## 3 Results

### 3.1 Patients characteristics

The mean age of the patients included was 62 years ± 13.0, with a range of 27–87 years. Table 1 provides a detailed overview of the demographic characteristics of the study sample, including age, sex, and other relevant factors. Of the patients, 161 were male and 78 females. The study sample included 155/239 (65%) with an Agatston score greater than 0.

**Table 1:** Patient demographics and image acquisition.

	Total	UHR	NRM
Variable	Value (n = 239)	Value (n = 66)	Value (n = 173)
Age (y) *	62 ± 13.0	67 ± 10.2	59.5 ± 12.9
Age range (y)	27–87		
Sex			
Male	161 (67)	52 (78.8)	109 (63)
Female	78 (33)	14 (21.2)	64 (37)
Heart rate (bpm) *	66.2 ± 11.2		
Arrythmias	44 (18)		
β-blocker use	153 (64)		
β-blocker dose (mg)*	10.0 ± 5.3		
Nitroglycerin use	225 (94)		
cCTA image acquisition			
UHR (total)	66 (28)		
UHR (Spiral)	12 (5)		
UHR (Sequence)	54 (23)		
NRM (total)	173		
NRM (Flash)	111		
NRM (Spiral)	24		
NRM (Sequence)	38		
Body mass index*	28.2 ± 5.9		
DLP (mGy·cm) (total)*	285.0 ± 278.81	332.91 ± 314.84	266.72 ± 4.8
DLP (mGy·cm) (CACS)*	36.32 ± 12.89	36.87 ± 11.89	36.11 ± 0.49

Data in parentheses are in percentages of numbers of patients.

cCTA (coronary CT angiography), UHR (ultra-high resolution), NRM (normal resolution mode), DLP (dose length product), CACS (coronary artery calcium scoring).

* Data are means ± SDs.

### 3.2 Qualitative reading

**Fig. 2:**
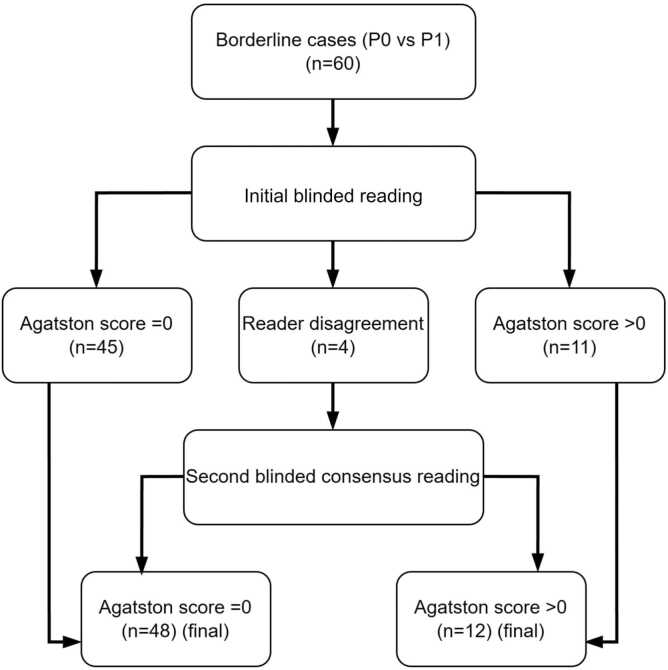
Flow Chart shows the process of the blinded reading of borderline cases.

Two radiologists re-evaluated 60 patients (25%) who had discrepancies between their TNC and VNC_PureCalcium_ Agatston scores, where one of the scores was zero. Of these 60 patients, the initial independent blinded review by two radiologists resulted in 45 (75%) being assigned an Agatston score of 0 and 11 (18%) a score greater than 0. For 4 patients (7%), the two radiologists disagreed. A subsequent consensus reading finalized the classification, resulting in 48 patients being assigned a score of 0 and 12 patients a score greater than 0. [see Fig. 2].

### 3.3 Comparison of agatston scores obtained from TNC and VNC_PureCalcium_ CACS

**Fig. 3:**
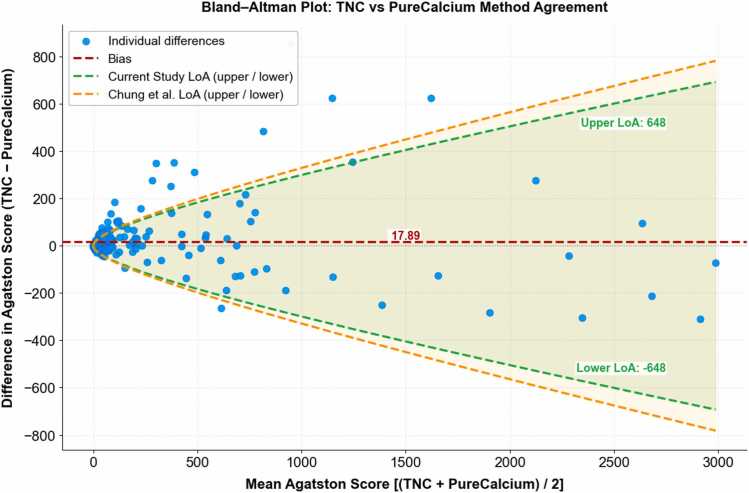
Bland-Altman plot illustrating agreement between coronary artery calcium scores derived from true non-contrast (TNC) and PureCalcium-based virtual non-contrast (VNC) images. Each blue dot represents an individual patient. The red dashed line indicates the mean bias (+17.89 Agatston units), reflecting a slight systematic underestimation by VNC_PureCalcium_ relative to TNC. The green dashed lines represent the 95% limits of agreement (LoA) of the current study (upper: +648; lower: −648), derived using quantile regression to account for heteroscedasticity. The orange dashed lines represent the reference repeatability limits defined by Chung et al. [9] for comparison.

**Fig. 4:**
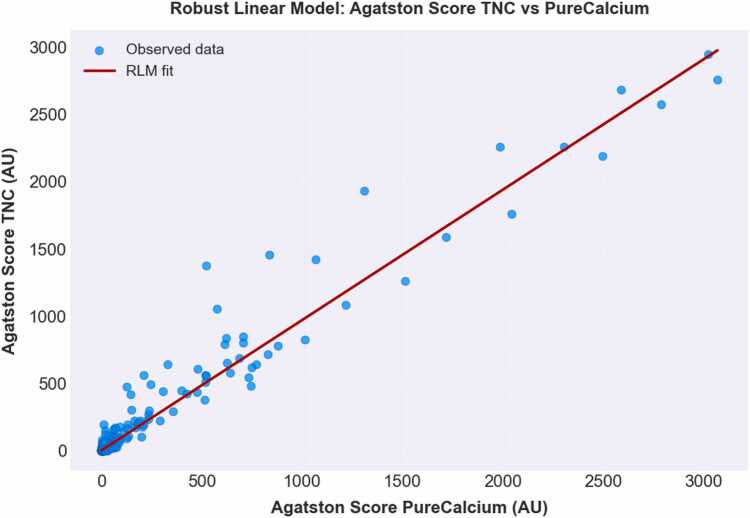
Scatterplot illustrates the distribution of coronary artery calcium scoring (CACS) obtained from true noncontrast (TNC) and PureCalcium-based virtual non-contrast (VNC) images. The regression line is displayed, with an intercept of 3.27 [95% CI: 1.28, 5.87] and a slope of 0.97 [95% CI: 0.97, 0.98]; *p* < 0.001).

The comparison of the Agatston scores derived from the TNC and VNC_PureCalcium_ imaging techniques showed no evidence of a difference in the median Agatston scores (14.2 [IQR (175.0), Q1-Q3, 0.0 – 175.0; range, 0.0 – 2951.2] vs 8.0 [IQR (124,8), Q1-Q3 1.8 – 126.6; range, 0.0 – 3069.3]; *p* = 0.54). The empirical equivalence test comparing VNC_PureCalcium_ and TNC images showed that 207 of the 239 (86.6%) VNC_PureCalcium_ measurements fell within the predefined equivalence limits shown in Fig. 3. The proportion of measurements outside the repeatability limit was 13.4%, indicating good agreement between methods. Table 2 provides an overview of the distribution of Agatston scores before qualitative reading, and Table 3 after. Furthermore, the correlation between the two methods was excellent (intraclass correlation coefficient, 0.97 [95% CI: 0.97, 0.98] indicating a high level of consistency in their measurements. Also, the robust linear regression analysis of TNC and VNC_PureCalcium_ CACS revealed a significant relationship between them (intercept = 3.27 [95% CI: 1.28, 5.87]; slope = 0.97 [95% CI: 0.97, 0.98*]; p* < 0.001), with a 0.97 (*β*) increase in TNC CACS for each point increase in VNC_PureCalcium_ CACS. [see Fig. 4].

**Table 2:** Distribution of Agatston Scores in TNC vs. PureCalcium images before qualitative reading.

Reconstruction Type and Agatston Score	Number of Cases
TNC images	
0	84
1–100	81
101–300	29
301–999	29
> 1000	16
Pure Calcium images	
0	42
1–100	133
101–300	27
301–999	23
> 1000	14

TNC= true non-contrast.

**Table 3:** Distribution of Agatston Scores in TNC vs. PureCalcium images after qualitative reading.

Reconstruction Type and Agatston Score	Number of Cases
TNC images	
0	86
1–100	79
101–300	29
301–999	29
> 1000	16
Pure Calcium images	
0	87
1–100	88
101–300	27
301–999	23
> 1000	14

TNC= true non-contrast.

The 95% limits of agreement from the quantile regression formula were estimated as:

*Y* = (0.14 × *M* + 5.01 × *M*(1/2) + 0.96), where *M* = (Agatston[TNC] + Agatston[VNC_PureCalcium_])/2.

**Fig. 5:**
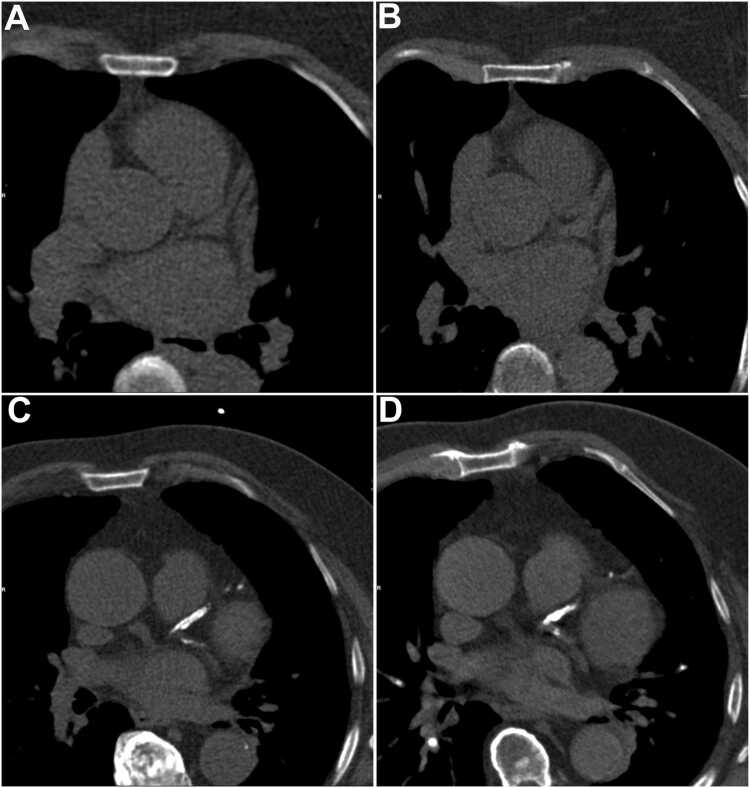
Images of coronary artery calcifications in true non-contrast (TNC) coronary artery calcium scoring (CACS) (left column) and PureCalcium-based virtual non-contrast (VNC) images from coronary CT angiography (CCTA) (right column). **(A-B)** shows an Image of a 58-year-old female, CAD-RADS P0. **(C-D)** shows an Image of an 87-year-old male, CAD-RADS P4, l ultra-high resolution (UHR) with spectral image data.

Eighteen (11.7%) of the observations were outside this repeatability limit. Considering Agatston scores of 50 and 200 for two sample patients using TNC images, the calculated 95% limits of agreement were found to be 7 – 93 and 100 – 300 Agatston units. In comparison, for repeated TNC measurements, the 95% limits of agreement were 5 – 94 and 94 – 305, respectively [9]. The Bland-Altman plot in Fig. 3 shows the 95% repeatability limits of our current study (green dotted line) in comparison to the standardized limits determined via Chung et al. [9] equations (yellow dotted line) and the distribution of findings (blue dots). A visual representationof the variation in Agatston scores can be found in Fig. 5.

### 3.4 Impact of differences in CACS on plaque burden category classification

There was evidence of a difference between the P classification of TNC and VNC_PureCalcium_ images before (*p* = 0.005) and after (*p* < 0.001) blinded qualitative readings (Wilcoxon signed-rank test)*.*

**Fig. 6:**
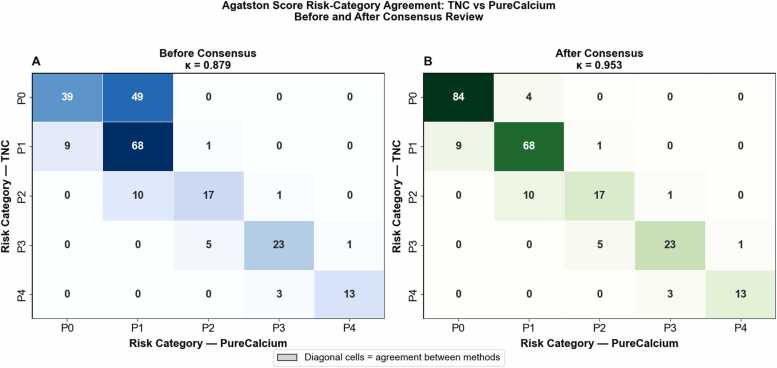
**A**: Plaque Classification based on Coronary Artery Disease Reporting and Data System (CAD-RADS) determined by the calculated Agatston Score using true non-contrast (TNC) and PureCalcium-based virtual non-constrast (VNC), before blinded reading. **B**: Plaque Classification based on Coronary Artery Disease Reporting and Data System (CAD-RADS) determined by the calculated Agatston Score using true non-contrast (TNC) and PureCalcium-based virtual non-contrast (VNC), after blinded reading in P0 and P1.

Prior to qualitative correction, classification was consistent in 160 (66.9%) patients, whereas in 79 patients, there was a discrepancy between VNC_PureCalcium_ and TNC P classifications. On VNC_PureCalcium_ images, 49 patients were misclassified as P1 instead of P0 on TNC images, 9 patients were misclassified as P0 instead of P1, and 10 patients were classified as P1 instead of P2 with a median Agatston score of 3.8 [see Fig. 6A].

Following blinded qualitative reading for borderline cases, classification consistency improved. The number of fully concordant classifications increased to 205 (85.8%) patients, with discrepancies reduced to 34 cases. Notably, misclassifications from P0 to P1 decreased from 49 to 4 cases, and the number of P0 to P0 matches increased from 37 to 84. However, despite this improvement, the Wilcoxon signed-rank test remained significant (*p* < 0.001), indicating residual systematic differences between the modalities (see Fig. 6B).

The categorical agreement of plaque classification between TNC CACS and VNC_PureCalcium_ CACS before blinded consensus reading was κ = 0.88. After an independent blinded review followed by the consensus adjudication of borderline cases, agreement improved substantially to κ = 0.95, reflecting almost perfect agreement.

### 3.5 Subgroup analysis

Table 4 provides a detailed overview of the CAD-RADS-Distribution after qualitative reading, separately for UHR and NRM.

**Table 4:** CAD-RADS-Distribution after qualitative reading.

CAD-RADS-Distribution	Number of Cases (UHR) (n = 66)	Number of Cases (NRM) (n = 173)
P0	0 (0)	88 (50.9)
P1	11 (16.7)	67 (38.7)
P2	17 (25.8)	11 (6.4)
P3	22 (33.3)	7 (4.0)
P4	16 (24.2)	0 (0)

Data in parentheses are in percentages of numbers of patients.

UHR (ultra-high resolution), NRM (normal resolution mode).

CAD-RADS = Coronary Artery Disease Reporting and Data System.

The median of the Agatston score for UHR was 478.4 [IQR (680.3) Q1-Q3, 164.9–845.2] and for NRM 0.1 [IQR (32.3) Q1-Q3, 0.0–32.3]. The proportion of measurements within the repeatability limit was 48 (72.7%) for UHR and 159 (91.9%) for NRM, indicating excellent agreement between methods.

The number of fully concordant classifications was 50 (75.8%) patients for UHR and 155 (89.6%) for NRM.

The categorical agreement of plaque classification between TNC CACS and VNC_PureCalcium_ CACS for UHR vs NRM after the consensus adjudication was κ = 0.89 vs 0.91, reflecting almost perfect agreement. The median for Agatston score agreement for UHR was 95.45 [IQR (148.1) Q1-Q3, 38.6–186.7] and for NRM 0.3 [IQR (9.2) Q1-Q3, 0.0–9.2], which is a relative difference of 19.95% for UHR, whereas for NRM the absolute differences were negligible, indicating excellent agreement.

### 3.6 Radiation dose analysis

During the examination, the radiation dose was measured using the dose-length product (DLP). The mean value of the total DLP was 285.0 ± 278.81 mGy·cm, while the DLP for the TNC scan was 36.32 ± 12.89 mGy·cm, which accounts for 19.15% ± 8.79 of the total DLP.

For UHR, only the total DLP was 332.91 ± 314.84 mGy·cm, and the DLP for the calcium scoring was 36.87 ± 11.89 mGy·cm. For NRM, the total DLP was 266.72 ± 4.8 mGy·cm, and the DLP for the TNC was 36.11 ± 0.49 mGy·cm.

## 4 Discussion

This study aimed to assess the reliability of the VNC_PureCalcium_ images derived from photon counting CCTA in a large, unselected patient cohort and to evaluate their performance in UHR imaging with spectral image data. Our results demonstrated that Agatston scores obtained from VNC_PureCalcium_ and TNC images showed no significant differences in the median values. Agreement between both methods was excellent with 88% of measurements falling within predefined equivalence limits and an intraclass correlation of 0.97, which indicates an excellent level of consistency, further supported by linear regression analysis. These results suggest that PureCalcium-based virtual non-contrast provides highly consistent calcium scoring while offering the potential to reduce radiation exposure by avoiding the non-contrast scan.

Despite the strong correlation of absolute scores, important differences were observed in plaque burden classification between true non-contrast and PureCalcium-based virtual non-contrast images. Especially patients with lower Agatston scores, were misclassified into higher or lower plaque categories. This subgroup is clinically the most relevant, as even small deviations in calcium burden may have a direct impact on therapy recommendations and cardiovascular risk stratifications. A MESA study from Budoff et al. [10] investigated the risk of coronary heart disease in patients with a minimal (score ≤10) or absent CAC. It demonstrated that asymptomatic patients with low or absent CAC have a very low risk of cardiovascular events in the future, whereas even minimal CAC (score 1–10) was associated with an approximately threefold increased cardiovascular risk.

After blinded consensus adjudication, the agreement of concordant plaque classifications increased substantially to 86%, leading to an almost perfect agreement (κ = 0.95) while residual systematic differences remained, with the Wilcoxon signed-rank test still showing significant differences (*p* < 0.005). The most clinically important finding of this study is that excellent numerical agreement in absolute Agatston scores does not translate into equivalent plaque burden classification. Despite an intraclass correlation of 0.97, classification agreement before adjudication was only 67%, rising to 86% after blinded consensus reading, underscoring that strong correlation between continuous scores cannot be equated with clinical interchangeability of plaque burden categories. The fact that 25% of the cohort required a second look highlights the current limitations of fully automated clinical implementation. Importantly, after blinded consensus reading, 75% of these borderline cases were reclassified with an Agatston score of 0, which shows that noise was potentially recognized as plaque. In only 4 patients did the two radiologists reach discordant conclusions, highlighting the modest interobserver variability in the assessment of borderline cases [Fig. 6B]. This improvement suggests that many discrepant cases reflected near-threshold instability rather than fundamental disagreement.

The present study extends previous work from Haag et al. [5]. With the newer VB10 software generation, it was possible to include a larger cohort, including patients with higher Agatston scores overall, due to the incorporation of UHR acquisition with spectral image data. In previous studies [3], [4], [5], [6], this subgroup was underrepresented due to technical limitations of prior software versions. This expands previous evidence for VNC_PureCalcium_ reliability across different patient populations and scan protocols. Our median total Agatston score was 14.2 [IQR 175.0, Q1-Q3 0.0–175.0] vs 4.8 [IQR 84.0, Q1-Q3 0.0–84.0]. With our maximum Agatston score reaching 2951.2 vs 2151.8. Comparing patients with an Agatston score of 0, Haag et al. [5] had an equal share of patients of 35% (84 vs. 59). In our study, 67% of all patients were correctly classified in plaque groups, whereby 33% were misclassified [Fig. 6A]. As in the study by Haag et al. [5], significantly more patients categorized as P1 were misclassified as P0 in VNC_PureCalcium_ CACS [Fig. 6A], highlighting the increased variability of borderline low-cases. This is further supported by a mean difference of + 17.89 [Fig. 3], indicating a slight underestimation by VNC_PureCalcium_ compared to TNC, especially in lower calcium burden. This demonstrates the most important clinical challenge to differentiate between P0 and P1 classifications. Similar to Emrich et al. [4] and Haag et al. [5], calcifications are either missed or image noise is misinterpreted as plaque, which artificially inflates Agatston scores and requires manual correction. Therefore, rounding values ≤ 0.9–0 was intended to reduce noise-related variability; however, this requires cautious interpretation given the established prognostic significance of even minimal CAC. Notably, Senoner et al. reported similar MACE rates in patients with CAC= 0 and CAC< 1, and values in this range fall outside the categories addressed by established classification systems such as CAD-RADS 2.0 [11], [12]. It should be noted that patients with known high calcification and prior treatment were preferentially assigned to UHR imaging, which resulted in higher median Agatston scores (478.4 (UHR) vs 0.1 (NRM) (see results and scan protocol)). In this context, the median absolute difference of 95.45 Agatston score units for UHR corresponds to a relative deviation of 19.95% of the median score, which appears acceptable given the elevated calcium burden and the preserved categorical agreement between methods.

UHR is only feasible in sequential and spiral scan modes due to technical limitations, potentially resulting in motion blurring in scans due to higher cardiac frequencies. A study from Risch et. al. [13] revealed that VNC-based CACS showed higher variability with rising heart rate [13]. Studies comparing conventional VNC imaging derived from dual-energy CT (DECT) [3], [14], [15], [16], showed similar results in feasibility and underestimation in lower coronary plaque burden. A study by Sartoretti et. al. [17] yielded comparable findings as Risch et. al. [13], that medium and high calcifications increased up to 50% at heart rates > 75/min. With the PureCalcium algorithm, it is now possible to generate UHR images with spectral image data. This allows to include patients with higher coronary calcium burden. This is an important technical advancement towards patients with advanced coronary calcification.

In contrast to NMR, UHR showed a slightly higher total radiation dose (332.91 ± 314.84 vs. 266.72 ± 4.8 mGy·cm). However, the clinically relevant radiation dose for CACS remained comparable (36.87 ± 11.89 vs.36.11 ± 0.49 mGy·cm) [Table 1], suggesting that the increased radiation dose was likely due to the UHR acquisition.

As in the study of Haag et al. [5], the proportion of radiation dose was similar (19.15% ± 8.79 vs. 19.7% ± 8.8), indicating the potential for radiation dose reduction. Fink et al. [6] provided a phantom study, comparing the radiation doses of TNC and VNC_PureCalcium_ on an older syngo.via Software (Version VB60; Siemens Healthineers). The results showed that VNC_PureCalcium_ could maintain equal image quality as TNC while reducing the radiation dose and blooming artifacts [6]. The study of Langenbach et. al. [18] investigated a reduction of radiation dose in dual-layer spectral-detector CT, allowing a detection and evaluation of calcification without the need for an additional TNC scan. Given the potential for reducing the radiation dose, the PureCalcium algorithm may seem promising in replacing the additional TNC scan.

Several limitations should be acknowledged. The retrospective nature of this study, as well as the use of a single-center sample size, implicates the presence of biases, leading to different results in regard to generalization and statistical power. Patients with known high calcium burden were preferentially assigned to UHR imaging, indicating a possible selection bias. Therefore, the observed differences between UHR and NMR may be influenced not only by differences in imaging performance, but also by the unequal distribution of complex cases between the subgroups. A potential subjective bias should be discussed. 4 Patients received consensus reading, so observer-dependent interpretation cannot be excluded. Due to technical limitations, the slice thickness of TNC and VNC_PureCalcium_ images was different (3 mm vs. 2 mm). This methodological difference represents an important limitation because slice thickness may affect calcium detectability and, consequently, plaque burden classification. In our retrospective study, a homogenization of reconstruction parameters was not possible. This potentially adds more noise to the images, leading to a higher misclassification. Nevertheless, the study of Haag et al. [5] showed positive results in regard to a strong correlation in Agatston scores between VNC_PureCaclium_ and TNC. UHR with spectral image data may be more sensitive to small calcifications, resulting in reconstruction-related variability (72.7% agreement vs. 91.9% in NRM). For image reconstruction, standard acquisition modes were used because of the retrospective nature of this study, which further limits the generalizability of the findings. Depending on experience, small calcifications may be considered as noise, showing the interobserver variability. Further research is needed to investigate the influence of different PureCalcium reconstruction settings and to validate the generalizability of these findings in prospective multicenter studies across different institutions, patient populations, and scanner implementations.

In summary, PureCalcium-based VNC derived from photon-counting CCTA demonstrated excellent agreement with TNC images for absolute Agatston scoring, with almost perfect agreement after blinded consensus adjudication. However, excellent agreement of continuous Agatston scores did not translate into equivalent plaque burden classification. The observed discrepancies, particularly in patients with low calcium burden and borderline P0/P1 classifications, indicate that PureCalcium-based VNC images cannot yet be considered fully interchangeable with TNC imaging for clinical risk categorization. Nevertheless, VNC_PureCalcium_ CACS shows potential in replacing true non-contrast CACS in patients with moderate or high calcium burden and could contribute to future radiation dose reduction. Additional technical refinements and prospective multicenter validation studies are required before PureCalcium-based virtual non-contrast coronary artery calcium scoring can replace true non-contrast coronary artery calcium scoring in routine clinical practice.

## CRediT authorship contribution statement

**Kroeger Jan:** Writing – review & editing, Visualization, Supervision. **Jan Borggrefe:** Writing – review & editing. **Gertz Roman:** Writing – review & editing. **Haag Nina:** Writing – review & editing. **Alexey Surov:** Writing – review & editing. **Niehoff Julius:** Writing – review & editing. **Iram Shahzadi:** Writing – review & editing, Formal analysis. **Sophia Engel:** Writing – original draft, Formal analysis, Conceptualization.

## Informed consent

Written informed consent was waived by the Institutional Review Board.

## Ethical approval

Institutional Review Board approval was obtained and the study was performed in accordance with the Declaration of Helsinki.

## Funding

This research did not receive any specific grant from funding agencies in the public, commercial, or not-for-profit sectors.

## Guarantor

The scientific guarantor of this publication is Jan Robert Kroeger.

## Study subjects or cohorts overlap

No study subjects or cohorts have been previously reported.

## Methodology

Methodology: retrospective, cross sectional study, performed at one institution

## Statistics and biometry

One of the authors (Iram Shahzadi) has significant statistical expertise.

## Declaration of Competing Interest

The authors of this manuscript declare relationships with the following companies:

The Ruhr University Bochum, Johannes Wesling Hospital, Department of Radiology, Neuroradiology and Nuclear Medicine, Germany has an institutional research Grant from Siemens Healthineers.

Iram Shahzadi and Nina P. Haag are former employees of Siemens Healthineers.

Roman J. Gertz is on the speaker’s bureau of Philips Healthcare and Guerbet GmbH.

Jan Borgrefe has received speaker honoraria from Siemens Healthineers and Philips Healthcare.

Jan Robert Kröger has received speaker honoraria from Siemens Healthineers and GE Healthcare.
